# Dentists’ knowledge and preference regarding gingival displacement methods

**DOI:** 10.1186/s12903-023-03218-1

**Published:** 2023-08-16

**Authors:** Islam Abd Alraheam, Susan Hattar, Aya Al-Asmar, Abeer Alhadidi, Saif Abu Hamour, Amira Aldroubi, Faleh A. Sawair

**Affiliations:** 1 Department of Restorative Dentistry, University of Jordan, Amman, Jordan; 2 Department of Oral and Maxillofacial Surgery, University of Jordan, Amman, Jordan; 3 Intern, Jordan University Hospital, Amman, Jordan

**Keywords:** Gingival displacement, Gingival retraction, Retraction cord, Retraction paste

## Abstract

**Introduction:**

An accurate impression is an essential procedure for fabricating indirect fixed restorations. To achieve a precise final impression, the management of gingival tissue is without doubt a crucial.

**Aim:**

To evaluate the use of different gingival displacement techniques among dental clinicians and to assess their associated knowledge and technique preferences.

**Methods:**

A self-designed survey was created electronically and sent to a list of dentists. The survey was composed of multiple sections. Participants who stated that they do not use GD methods were asked to answer the survey questions based on their knowledge. Descriptive statistics were generated, andChi-square test was used to examine the association between the different variables.

**Results:**

A total of 188 dentists participated in this study. The majority 144 (76.6%) use GD in their practice. When asked which technique yields a more accurate impression with lower incidence of repeating the impression, 93 (64.6%) reported retraction cord technique with a hemostatic agent results in a higher impression accuracy, while only 14 (9.7%) declared the retraction paste technique as being more accurate.

**Conclusion:**

The cordless GD technique is believed to be easier, faster, and less traumatic to the gingival tissues, nevertheless, the outcome of dental impressions is believed to be more predictable with the use of conventional retraction cords and hemostatic medicaments.

**Supplementary Information:**

The online version contains supplementary material available at 10.1186/s12903-023-03218-1.

## Introduction

An accurate impression is of paramount importance when fabricating indirect fixed restorations and plays a crucial role to ensure the final success rate of the prostheses [1–3]. To achieve an accurate dental impression, management of gingival tissue is mandatory, especially in challenging cases where the finish line is located equigingival or subgingival [2–5].

To achieve optimal result, the impression should have adequate thickness to prevent the tearing of the material once the impression is removed. This can be achieved by having proper finish line details associated with adequate gingival management [1, 2, 6]. Inaccurate impressions can cause severalproblems, such as misfitting of the final restoration and compromised marginal integrity leading to plaque accumulation. It has been shown that plaque accumulation is the main causative factor for gingival inflammation and caries, which might subsequently result in failure of the restoration or even lead to extraction of the tooth [1, 4, 6–8].

Gingival displacement (GD) is defined as the deflection of the marginal gingiva away from the tooth [3, 9]. This displacement creates both lateral and vertical spaces that are primordial for the exposure of subgingival margins, in addition to ensuring adequate bulk of injected impression material in the expanded gingival crevice. A sulcular width of at least 0.2 mm is mandatory to prevent tearing of the impression materials [10]. Furthermore, GD helps in controlling hemorrhage and achieving hemostasis to facilitate proper isolation required for hydrophobic impression materials as well as adhesive restorations [2, 3]. An ideal GD technique should retract the gingiva temporarily and atraumatically while achieving adequate homeostasis [2]. Clinicians have been using a variety of techniques for GD, categorized as mechanical, chemical, surgical, or a combination of the aforementioned.

The mechanical technique involves the use of retraction cords to displace gingival tissues. Retraction cords can either be used alone or in combination with hemostatic or vasoconstrictor agents to achieve adequate hemostasis [11, 12]. The use of retraction cords impregnated with a hemostatic medicament is considered one of the most used methods for gingival displacement [6]. From another perspective, in addition to being a time-consuming procedure, the use of retraction cords can sometimes cause gingival bleeding and patient discomfort especially when placed without local anesthesia [3, 11, 12]. Furthermore, improper placement of retraction cords might result in trauma and/or gingival recession, reflecting on the final prosthetic outcome [13, 14]. Nevertheless, the placement of retraction cords represents an inexpensive, simple, and widely used technique for gingival displacement [1, 4, 15, 16]. Retraction cords comes in different forms; twisted, braided, and knitted and depending on the clinical situation, it may be applied as a single- or double-cord technique [2].

Retraction cords impregnated with hemostatic chemicals help with achieving temporary local hemostatic effect. Unfortunately, these chemical substances tend to react with some impression materials and cause unfavorable side effects such gingival irritability and discoloration [11]. It has been shown that the use of epinephrine as a GD medicament can cause significant systemic side effects [2, 17]. Moreover, epinephrine is contraindicated in patients suffering from cardiovascular diseases such as hypertension, hyperthyroidism, and diabetes; therefore, it is not recommended to be used routinely in dental practice [2]. Other types of hemostatic agents like aluminum sulfate, aluminum potassium sulfate, aluminum chloride, and ferric sulfate are considered valuable alternative agents to be applied when more hemostasis is required. These topical agents are indeed considered clinically safe as they do not cause significant systemic side effects [2, 4, 17].

Recently, chemical retraction techniques were introduced in an attempt to overcome the disadvantages of conventional retraction cords [1, 11, 15]. The application of retraction paste is considered a less traumatic technique to achieve satisfactory gingival displacement [4, 12]. Currently, many materials are present in the market; one widely used is Expasyl Paste (KerrCorp, Orange, CA), which consists of kaolin and aluminum chloride. This material depends on the hygroscopic expansion of kaolin that occurs upon contact with the crevicular fluid, combined with the hemostatic activity of aluminum chloride, the resulting gingival displacement occurs in 2–4 min according to the manufacturer [3]. Magic Foam Cord (Coltene Whaledent AG, Altstatten, Switzerland) is another material that uses polyvinyl siloxane as an expanding medium in conjunction with the mechanical pressure exerted by Compre-Caps, to achieve gingival retraction [15]. Traxodent Hemodent Paste (Premier Dental Company, Plymouth Meeting, PA) is also comprised of 15% aluminum chloride topical paste along with cotton caps [15]. In summary, cordless techniques, while causing less discomfort to the patient, are considered less invasive and less time-consuming when compared to conventional retraction cords [4, 12, 18]. From another view since retraction paste systems depend on their expansion property upon contact with crevicular fluids, they might not provide enough displacement especially in cases of deep sulcus depth [4, 12, 19]. A randomized clinical trial investigated the GD using three different paste systems and reported a mean sulcular gingival width of (0.644 ± 0.22) in the Traxodent group, followed by the Expasyl group (0.590 ± 0.11), and the Magic Foam Cord group (0.528 ± 0.01) [20].

Regardless of all the attempts to compare the efficiency of gingival cords and paste systems, it is still believed that there is no technique with a superior success rate, and the choice of technique depends on the clinician’s preference [1, 4, 11, 13, 15]. Reviewing the literature does not reveal any evidence concerning the knowledge and preference regarding mechanical GD methods among dentists. This study was conducted with the aim of evaluating the use of two different techniques among a group of dental clinicians and exploring their associated knowledge and technique preferences.

## Methods

### Data collection

The study protocol was reviewed and approved by the Institutional Review Board of Jordan University Hospital (Ref #38–2022).

A self-designed survey was created electronically and emailed to a list of dentists obtained from the Jordanian Dental Association. The survey was also distributed via social media to Jordanian dentists who are members of various social media platforms. Personal identifiers were not used in the online questionnaire to maintain anonymity. The introduction of the questionnaire defined the study's purpose and objectives. The authors also stated unequivocally that participation is entirely voluntary, with no consequences for refusal or withdrawal. Responding to the questionnaire implied consent.

The survey was composed of multiple sections. The demographic section covered gender, cumulative GPA, years of experience, type of practice, and education level. The other sections assessed the knowledge and preference of dentists regarding the use of gingival retraction cords, types of cords, hemostatic medicaments, gingival retraction pastes, side effects, and other relevant factors as shown in Additional file 1. Participants who stated that they do not use GD methods were asked to answer the survey questions based on their knowledge.

## Statistical analysis

Statistical analysis was performed using SPSS for Windows release 16.0 (SPSS Inc., Chicago, IL, USA). Descriptive statistics were generated, and the Chi-square test and Fishers’ exact test were used to examine associations between the different variables. The significance level was set at *P* < 0.05.

## Results

A total of 188 dentists participated in this study. Their sociodemographic characteristics are shown in Table 1. The majority were females, general practitioners and specialists, with very good GPAs, clinical experience of more than 10 years, and working in the private sector. The associations between the sociodemographic variables and dentists’ knowledge and preference regarding the use of GD techniques based on their experience are shown in Table 2. The responses based on the dentist’s knowledge are shown in Table 3.

**Table 1:** The sociodemographic characteristics of the studied sample

Variable	Number (%)
Gender	
Male	80 (42.6)
Female	108 (57.4)
Education	
Intern	31 (16.5)
General Practitioner	68 (36.2)
Postgraduate student/ resident	19 (10.1)
Specialist	70 (37.2)
Cumulative GPA	
Excellent	40 (21.3)
Very good	79 (42.0)
Good	60 (31.9)
Fair	9 (4.8)
Experience (years)	
< 5	75 (39.9)
5–10	20 (10.6)
> 10	93 (49.5)
Work sector	
Academic	39 (20.7)
Private	87 (46.3)
Both	43 (22.9)
Not working	19 (10.1)

**Table 2:** The association between the sociodemographic variables and dentists’ knowledge and preference regarding GD techniques use based on their experience

		No. (%)	Gender		Education				GPA				Experience (years)			Work sector			
			M	F	Intern	GP	PGS	Spec	Exc	VG	G	F	< 5	5–10	> 10	Acad	Priv	Both	Not
Do you use any type of GD in your clinic?	Yes	144 (76.6)	83.8	71.3	61.3	76.5	78.9	82.9	75	77.2	78.3	66.7	68	75	83.9	66.7	82.8	81.4	57.9
	*P* value		**0.046**		0.13				0.88				0.053			0.051			
Use GD with:	Full coverage	130 (90.3)	92.5	88.3	100	86.5	86.7	91.4	93.3	90.2	91.5	66.7	94.1	80	89.7	88.5	91.7	85.7	100
	*P* value		0.39		0.37				0.40				0.26			0.52			
	Partial coverage	75 (53.6)	51.5	55.4	42.1	42.3	53.8	67.9	65.5	50.8	52.2	33.3	38.8	73.3	59.2	50	47.1	69.7	54.5
	*P* value		0.65		**0.041**				0.41				**0.022**			0.19			
	Composite	96 (68.6)	66.7	70.3	68.4	67.3	61.5	71.4	65.5	74.6	63	66.7	71.4	66.7	67.1	61.5	68.6	72.7	72.7
	*P* value		0.65		0.91				0.62				0.87			0.81			
	Impression for implant	34 (24.3)	27.3	21.6	26.3	30.8	15.4	19.6	20.7	22	28.3	33.3	22.4	26.7	25	19.2	17.1	33.3	54.5
	*P* value		0.44		0.49				0.79				0.92			**0.026**			
Types of impressions that you use GD with it/them?	Digital	2 (1.4)	3	0	5.3	0	0	1.7	3.3	0	2.1	0	2	0	1.3	0	1.4	2.9	0
	Conventional	70 (48.6)	43.3	53.2	52.6	57.7	40	41.4	40	49.2	51.1	66.7	54.9	46.7	44.9	61.5	48.6	34.3	63.6
	Both	72 (50)	53.7	46.8	42.1	42.3	60	56.9	56.7	50.8	46.8	33.3	43.1	53.3	53.8	38.5	50	62.9	36.4
	*P* value		0.19		0.37				0.74				0.79			0.40			
In case of fixed prostheses, do you use GD during:	Preparation	62 (43.1)	53.7	33.8	57.9	44.2	26.7	41.4	43.3	41	42.6	66.7	51	46.7	37.2	42.3	48.6	28.6	54.5
	*P* value		**0.016**		0.33				0.69				0.29			0.21			
	Impression	135 (93.8)	92.5	94.8	94.7	92.3	86.7	96.6	90	96.7	95.7	66.7	96.1	86.7	93.6	92.3	95.8	88.6	100
	*P* value		0.58		0.52				0.11				0.47			0.33			
	Provisional -	25 (17.4)	26.9	9.1	21.1	19.2	13.3	15.5	10	23	14.9	16.7	15.7	20	17.9	19.2	15.3	22.9	9.1
	*P* value		**0.005**		0.89				0.45				0.91			0.67			
	Cementation	39 (27.1)	32.8	22.1	31.6	15.4	26.7	36.2	43.3	23	25.5	0	19.6	20	33.3	26.9	23.6	34.3	27.3
	*P* value		0.15		0.10				0.045				0.19			0.72			
Which type of GD technique do you use?	Retraction cord	104 (72.2)	70.1	74	94.7	76.9	93.3	55.2	60	70.5	78.7	100	94.1	60	60.3	73.1	72.2	62.9	100
	Cordless (retraction paste)	4 (2.8)	3	2.6	0	1.9	0	5.2	3.3	4.9	0	0	0	0	5.1	0	4.2	2.9	0
	Cord and paste	36 (25)	26.9	23.4	5.3	21.2	6.7	39.7	36.7	24.6	21.3	0	5.9	40	34.6	26.9	23.6	34.3	0
	*P* value		0.87		**0.003**				0.28				** < 0.001**			0.30			
What is the technique of retraction cord do you use?	Single technique	37 (25.7)	25.4	26	36.8	32.7	13.3	19	16.7	24.6	34	16.7	29.4	13.3	25.6	11.5	26.4	28.6	45.5
	Double technique	10 (6.9)	7.5	6.5	21.1	0	0	10.3	10	4.9	8.5	0	7.8	0	7.7	23.1	4.2	2.9	0
	Both (depends on the case)	97 (67.4)	67.2	67.5	42.1	67.3	86.7	70.7	73.3	70.5	57.4	83.3	62.7	86.7	66.7	65.4	69.4	68.6	54.5
	*P* value		0.97		**0.007**				0.55				0.51			**0.03**			
What is the technique of retraction cord do you use?	Twisted	43 (29.9)	35.8	24.7	10.5	40.4	26.7	27.6	26.7	23	40.4	33.3	17.6	46.7	34.6	15.4	33.3	31.4	36.4
	*P* value		0.15		0.10				0.25				**0.035**			0.31			
	Knitted	39 (27.1)	29.9	24.7	21.1	21.2	20	36.2	33.3	27.9	21.3	33.3	23.5	46.7	25.6	42.3	13.9	40	36.4
	*P* value		0.49		0.26				0.67				0.22			**0.004**			
	Braided	47 (32.6)	31.3	33.6	26.3	15.4	40	48.3	53.3	34.4	17	33.3	29.4	33.3	34.6	42.3	31.9	28.6	27.3
	*P* value		0.76		**0.003**				**0.01**				0.83			0.68			
Do you use impregnated retraction cord?	Yes	83 (57.6)	65.7	50.6	31.6	42.3	66.7	77.6	76.7	60.7	44.7	33.3	41.2	66.7	66.7	73.1	50	65.7	45.5
	*P* value		0.16		**0.001**				** < 0.001**				**0.042**			0.34			
Do you soak the retraction cord in a hemostatic medicament before you pack it?	Yes	90 (62.5)	62.7	62.3	73.7	55.8	86.7	58.6	70	60.7	59.6	66.7	72.5	60	56.4	88.5	52.8	65.7	54.5
	*P* value		0.97		0.11				0.80				0.18			**0.013**			
What is/are the type/s of the hemostatic medicament that you use with the retraction cord?	Epinephrine	8 (5.6)	7.5	3.9	5.3	3.8	13.3	5.2	6.7	8.2	0	16.7	5.9	20	2.6	7.7	4.2	8.6	0
	*P* value		0.35		0.56				0.17				**0.07**			0.63			
	Aluminum potassium sulfate	27 (18.8)	28.4	10.4	0	11.5	13.3	32.8	26.7	21.3	10.6	16.7	3.9	13.3	29.5	15.4	15.3	34.3	0
	*P* value		**0.006**		**0.001**				0.32				** < 0.001**			**0.018**			
	Aluminum chloride	36 (25)	31.3	19.5	26.3	21.2	26.7	27.6	26.7	26.2	19.1	50	19.6	40	25.6	30.8	15.3	34.3	45.5
	*P* value		0.12		0.88				0.40				0.27			0.043			
	Aluminum Sulfate	43 (29.9)	28.4	31.2	26.3	25	20	37.9	50	24.6	23.4	33.3	25.5	46.7	29.5	30.8	27.8	31.4	36.4
	*P* value		0.71		0.36				**0.06**				0.31			0.94			
	Ferric Sulfate	58 (40.3)	35.8	44.2	52.6	42.3	53.3	31	33.3	39.3	44.7	50	54.9	33.3	32.1	34.6	34.7	48.6	63.6
	*P* value		0.31		0.22				0.74				**0.011**			0.19			
	Local anesthesia	15 (10.4)	6	14.3	10.5	11.5	6.7	10.3	10	8.2	14.9	0	11.8	6.7	10.3	15.4	11.1	8.6	0
	*P* value		0.10		0.96				0.57				0.85			0.55			
Have you experienced any adverse systemic problems using epinephrine as a hemostatic agent?	Yes	12 (8.3)	11.9	5.2	5.3	11.5	6.7	6.9	10	9.8	6.4	0	5.9	20	7.7	11.5	4.2	17.1	0
	*P* value		0.32		0.13				0.59				0.26			**0.01**			
Have you experienced any adverse local tissue problems when using a hemostatic medicament?	Yes	44 (30.6)	41.8	20.8	26.3	25	33.3	36.2	43.3	23	29.8	50	21.6	60	30.8	38.5	26.4	37.1	18.2
	*P* value		0.24		0.34				0.48				**0.06**			0.24			
Did the hemostatic medicament cause any problem or affect the impression material?	Yes	23 (16)	22.4	10.4	5.3	9.6	26.7	22.4	26.7	16.4	8.5	16.7	11.8	33.3	15.4	19.2	9.7	28.6	9.1
	*P* value		0.11		0.20				0.28				0.36			0.15			
Do you use electrosurgery in your clinic to obtain GD and hemostasis?	Yes	38 (26.4)	35.8	18.2	5.3	21.2	20	39.7	20	31.1	21.3	50	11.8	46.7	32.1	15.4	27.8	34.3	18.2
	*P* value		**0.017**		**0.014**				0.30				**0.005**			0.36			
What is/are the type/s of retraction paste that you use?	Expasyl	24 (16.7)	17.9	15.6	10.5	11.5	13.3	24.1	36.7	13.1	8.5	16.7	9.8	33.3	17.9	23.1	15.3	20	0
	*P* value		0.71		0.27				**0.017**				0.28			0.34			
	Racegel	9 (6.2	9	3.9	5.3	7.7	13.3	3.4	3.3	8.2	4.3	16.7	3.9	13.3	6.4	7.7	4.2	11.4	0
	*P* value		0.30		0.52				0.52				0.42			0.40			
	Traxodent	7 (4.9)	9	1.3	0	3.8	13.3	5.2	6.7	4.9	2.1	16.7	2	13.3	5.1	0	2.8	14.3	0
	*P* value		0.05		0.33				0.43				0.20			**0.029**			
	GingiTrac	4 (2.8)	4.5	1.3	0	1.9	13.3	1.7	3.3	3.3	0	16.7	2	13.3	1.3	3.8	0	8.6	0
	*P* value		0.34		0.19				0.19				0.68			0.08			
	Access Edge	4 (2.8)	4.5	1.3	0	1.9	13.3	1.7	3.3	3.3	0	16.7	2	13.3	1.3	3.8	0	8.6	0
	*P* value		0.34		0.19				0.19				0.68			0.08			
	Astringent Retraction Paste	30 (20.8)	25.4	16.9	5.3	19.2	20	27.6	23.3	21.3	19.1	16.7	5.9	33.3	28.2	11.5	19.4	37.1	0
	*P* value		0.31		0.45				0.27				0.17			0.58			
Which technique yields more accurate impression and less incidence of repeating the impression?	Retraction cords with hemostatic agent	93 (64.6)	70.1	59.7	73.7	53.8	80	67.2	66.7	62.3	63.8	83.3	62.7	60	66.7	80.8	54.2	68.6	81.8
	Retraction cords without hemostatic agent	17 (11.8)	10.4	13	5.3	15.4	6.7	12.1	16.7	9.8	12.8	0	11.8	20	10.3	3.8	16.7	8.6	9.1
	Retraction paste	14 (9.7)	9	10.4	0	9.6	6.7	13.8	10	13.1	6.4	0	3.9	20	11.5	7.7	11.1	8.6	9.1
	No difference	20 (13.9)	10.4	16.9	21.4	21.2	6.7	6.9	6.7	14.8	17	16.7	21.6	0	11.5	7.7	18.1	14.3	0
	*P* value		0.59		0.23				0.81				0.15			0.39			
When you do GD with non-vital tooth, do you use local anesthesia with:	Retraction cord	96 (66.7)	65.7	67.5	78.9	71.2	40	65.5	56.7	68.9	70.2	66.7	70.6	80	61.5	61.5	69.4	60	81.8
	Retraction paste	3 (2.1)	3	1.3	0	0	0	5.2	3.3	3.3	0	0	0	0	3.8	3.8	1.4	0	9.1
	Both	15 (10.4)	11.9	9.1	5.3	7.7	20	12.1	13.3	8.2	10.6	16.7	7.8	6.7	12.8	7.7	9.7	17.1	0
	Neither	30 (20.8)	19.4	22.1	15.8	21.2	40	17.2	26.7	19.7	19.1	16.7	21.6	13.3	21.8	26.9	19.4	22.9	9.1
	*P* value		0.82		0.20				0.93				0.57			0.45			
Retraction paste is easier to use than retraction cord:	Strongly agree	12 (8.3)	6	10.4	10.5	7.7	6.7	8.6	10	4.9	12.8	0	7.8	6.7	9	7.7	9.7	5.7	9.1
	Agree	63 (43.8)	46.3	41.6	42.1	44.2	46.7	43.1	43.3	50.8	36.2	33.3	45.1	46.7	42.3	57.7	33.3	54.3	45.5
	Neither agree nor disagree	58 (40.3)	37.3	42.9	42.1	40.4	40	39.7	36.7	41	40.4	50	39.2	40	41	34.6	51.4	25.7	27.3
	Disagree	9 (6.2)	9	3.9	5.3	5.8	6.7	6.9	3.3	3.3	10.6	16.7	7.8	6.7	5.1	0	4.2	11.4	18.2
	Strongly disagree	2 (1.4)	1.5	1.3	0	1.9	0	1.7	6.7	0	0	0	0	0	2.6	0	1.4	2.9	0
	*P* value		0.60		0.81				0.21				0.97			0.18			
Retraction paste is less time consuming than retraction cord:	Strongly agree	13 (9)	10.4	7.8	15.8	5.8	6.7	10.3	10	6.6	12.8	0	9.8	0	10.3	7.7	9.7	8.6	9.1
	Agree	79 (54.9)	52.2	57.1	57.9	48.1	66.7	56.9	50	65.6	46.8	33.3	56.9	73.3	50	69.2	50	57.1	45.5
	Neither agree nor disagree	43 (29.9)	32.8	27.3	21.1	38.5	26.7	25.9	30	24.6	34	50	27.5	26.7	32.1	19.2	36.1	25.7	27.3
	Disagree	7 (4.9)	3	6.5	5.3	5.8	0	5.2	3.3	3.3	6.4	16.7	5.9	0	5.1	3.8	2.8	5.7	18.2
	Strongly disagree	2 (1.4)	1.5	1.3	0	1.9	0	1.7	6.7	0	0	0	0	0	2.6	0	1.4	2.9	0
	*P* value		0.79		0.90				0.20				0.69			0.65			
Retraction paste is more comfortable to the patient than retraction cord:	Strongly agree	13 (9)	10.4	7.8	10.5	5.8	13.3	10.3	10	8.2	8.5	16.7	5.9	26.7	7.7	7.7	6.9	11.4	18.2
	Agree	74 (51.4)	49.3	53.2	57.9	40.4	60	56.9	60	62.3	36.2	16.7	51	46.7	52.6	73.1	41.7	54.3	54.5
	Neither agree nor disagree	49 (34)	35.8	32.5	31.6	42.3	26.7	29.3	23.3	27.9	44.7	66.7	37.3	20	34.6	19.2	44.4	25.7	27.3
	Disagree	7 (4.9)	4.5	5.2	0	9.6	0	3.4	3.3	1.6	10.6	0	5.9	6.7	3.8	0	5.6	8.6	0
	Strongly disagree	1 (0.7)	0	1.3	0	1.9	0	0	3.3	0	0	0	0	0	1.3	0	1.4	0	0
	*P* value		0.84		0.55				0.084				0.41			0.29			
Retraction paste is more effective to control bleeding than retraction cord:	Strongly agree	2 (1.4)	1.5	1.3	0	0	0	3.4	3.3	1.6	0	0	0	0	2.6	0	0	2.9	9.1
	Agree	42 (29.2)	26.9	31.2	36.8	26.9	26.7	29.3	23.3	39.3	21.3	16.7	29.4	26.7	29.5	38.5	25	37.1	9.1
	Neither agree nor disagree	70 (48.6)	47.8	49.4	63.2	51.9	46.7	41.4	50	42.6	51.1	83.3	54.9	46.7	44.9	42.3	51.4	42.9	63.6
	Disagree	29 (20.1)	23.9	16.9	0	19.2	26.7	25.9	20	16.4	27.7	0	15.7	26.7	21.8	19.2	22.2	17.1	18.2
	Strongly disagree	1 (0.7)	0	1.3	0	1.9	0	0	3.3	0	0	0	0	0	1.3	0	1.4	0	0
	*P* value		0.74		0.45				0.29				0.84			0.41			
Retraction paste is less traumatic to the gingival tissue and cause less recession than retraction cord:	Strongly agree	16 (11.1)	11.9	10.4	5.3	13.5	6.7	12.1	10	9.8	14.9	0	11.8	20	9	7.7	12.5	5.7	27.3
	Agree	81 (56.2)	55.2	57.1	84.2	46.2	60	55.2	56.7	62.3	44.7	83.3	62.7	46.7	53.8	65.4	51.4	54.3	72.7
	Neither agree nor disagree	36 (25)	23.9	26	10.5	34.6	20	22.4	26.7	18	34	16.7	21.6	20	28.2	19.2	33.3	20	0
	Disagree	9 (6.2)	7.5	5.2	0	5.8	13.3	6.9	6.7	6.6	6.4	0	3.9	13.3	6.4	3.8	2.8	17.1	0
	Strongly disagree	2 (1.4)	1.5	1.3	0	0	0	3.4	0	3.3	0	0	0	0	2.6	3.8	0	2.9	0
	*P* value		0.98		0.30				0.64				0.63			**0.033**			
Retraction paste is more cost efficient than retraction cord:	Strongly agree	5 (3.5)	4.5	2.6	0	3.8	0	5.2	6.7	1.6	4.3	0	0	0	6.4	7.7	2.8	2.9	0
	Agree	25 (17.4)	14.9	19.5	0	21.2	33.3	15.5	6.7	19.7	19.1	33.3	9.8	20	21.8	11.5	18.1	25.7	0
	Neither agree nor disagree	67 (46.5)	40.3	51.9	73.7	42.3	53.3	39.7	43.3	42.6	55.3	33.3	60.8	26.7	41	50	43.1	40	81.8
	Disagree	41 (28.5)	37.3	20.8	26.3	28.8	13.3	32.8	33.3	34.4	17	33.3	27.5	46.7	25.6	26.9	29.2	31.4	18.2
	Strongly disagree	6 (4.2)	3	5.2	0	3.8	0	6.9	10	1.6	4.3	0	2	6.7	5.1	3.8	6.9	0	0
	*P* value		0.23		0.20				0.37				0.17			0.37			
Retraction paste causes gingival discoloration:	Strongly agree	3 (2.1)	3	1.3	0	0	6.7	3.4	3.3	1.6	2.1	0	2	0	2.6	3.8	1.4	2.9	0
	Agree	31 (21.5)	20.9	22.1	21.1	19.2	13.3	25.9	33.3	19.7	17	16.7	25.5	13.3	20.5	26.9	12.5	28.6	45.5
	Neither agree nor disagree	74 (51.4)	49.3	53.2	68.4	53.8	53.3	43.1	43.3	49.2	57.4	66.7	58.8	46.7	47.4	57.7	54.2	42.9	45.5
	Disagree	32 (22.2)	22.4	22.1	10.5	23.1	26.7	24.1	20	24.6	21.3	16.7	11.8	40	25.6	11.5	29.2	22.9	0
	Strongly disagree	4 (2.8)	4.5	1.3	0	3.8	0	3.4	0	4.9	2.1	0	2	0	3.8	0	2.8	2.9	9.1
	*P* value		0.75		0.69				0.89				0.43			0.18

**Table 3:** The association between the sociodemographic variables and dentists’ knowledge and preference regarding GD techniques use based on their knowledge

What is/are the type/s of restorations that you need to use GD technique with it/them?	Full coverage indirect restorations	37 (84.1)	84.6	83.9	83.3	87.5	75	83.3	100	72.2	92.3	66.7	91.7	100	66.7	69.2	93.3	75	100
	*P* value		0.95		0.94				0.16				0.068			0.16			
	Partial coverage indirect restoration	13 (29.5)	30.8	29	41.7	37.5	25	8.3	30	27.8	38.5	0	41.7	20	13.3	23.1	40	12.5	37.5
	*P* value		0.91		0.27				0.62				0.15			0.49			
	Composite direct restoration	21 (47.7)	30.8	54.8	75	43.8	50	25	50	61.1	38.5	0	66.7	40	20	30.8	53.3	50	62.5
	*P* value		0.15		0.10				0.21				**0.017**			0.49			
	Impression for implant	16 (36.4)	38.5	35.5	41.7	37.5	50	25	20	38.9	53.8	0	41.7	0	40	30.8	40	37.5	37.5
	*P* value		0.85		0.77				0.20				0.20			0.97			
What is the type of impression that you need to use GD techniques with it?	Digital impression	3 (6.8)	0	9.7	8.3	6.2	25	0	0	16.7	0	0	4.2	0	13.3	15.4	6.7	0	0
	Conventional impression	18 (40.9)	53.8	35.5	41.7	43.8	25	41.7	40	44.4	38.5	33.3	37.5	40	46.7	46.2	33.3	37.5	50
	Both	23 (52.3)	46.2	54.8	50	50	50	58.3	60	38.9	61.5	66.7	58.3	60	40	38.5	60	62.5	50
	*P* value		0.34		0.78				0.48				0.67			0.71			
What is/are the type/s of GD technique that you can use it/them?	Retraction cord	40 (90.9)	100	87.1	100	81.2	100	91.7	100	88.9	84.6	100	100	80	80	76.9	93.3	100	100
	*P* value		0.17		0.33				0.57				0.07			0.19			
	Cordless (retraction paste)	17 (38.6)	23.1	45.2	50	56.2	0	16.7	50	33.3	38.5	33.3	50	20	26.7	30.8	33.3	37.5	62.5
	*P* value		0.17		**0.025**				0.85				0.23			0.49			
	Electrosurgery	10 (22.7)	15.4	25.8	33.3	25	0	16.7	20	22.2	23.1	33.3	29.2	0	20	23.1	20	12.5	37.5
	*P* value		0.45		0.52				0.97				0.35			0.67			
Regarding the retraction cord techniques (single vs double), which technique is more accurate?	Single technique	5 (11.4)	15.4	9.7	8.3	6.2	25	16.7	0	16.7	7.7	33.3	4.2	20	20	23.1	13.3	0	0
	Double technique	13 (29.5)	23.1	32.3	41.7	31.2	0	25	40	27.8	30.8	0	37.5	40	13.3	30.8	13.3	37.5	50
	Depends on the case	17 (38.6)	30.8	41.9	41.7	56.2	75	0	40	44.4	30.8	33.3	45.8	0	40	23.1	46.7	37.5	50
	I do not know the difference between them	9 (20.5)	30.8	16.1	8.3	6.2	0	58.3	20	11.1	30.8	33.3	12.5	40	26.7	23.1	26.7	25	0
	*P* value		0.62		**0.002**				0.70				0.19			0.40			
Regarding the retraction cord types (impregnated vs non-impregnated), which technique is better?	Impregnated	18 (40.9)	30.8	45.2	41.7	31.2	75	41.7	40	50	30.8	33.3	33.3	20	60	53.8	40	50	12.5
	Non-impregnated	2 (4.5)	7.7	3.2	8.3	6.2	0	0	10	0	7.7	0	8.3	0	0	0	0	0	25
	Depends on the case	15 (34.1)	30.8	35.5	33.3	43.8	25	25	30	27.8	53.8	0	41.7	40	20	23.1	46.7	25	37.5
	I do not know the difference	9 (20.5)	30.8	16.1	16.7	18.8	0	33.3	20	22.2	7.7	66.7	16.7	40	20	23.1	13.3	25	25
	*P* value		0.60		0.79				0.38				0.39			0.15			
Are there any adverse systemic side effects that can happen when using epinephrine as a hemostatic agent?	Yes	20 (45.5)	38.5	48.4	58.3	43.8	25	41.7	20	44.4	61.5	66.7	45.8	60	40	53.8	46.7	37.5	37.5
	*P* value		0.55		0.67				0.21				0.74			0.85			
Are there any adverse local tissue problems that can happen when using a hemostatic medicament?	Yes	33 (75)	53.8	83.9	75	81.2	75	66.7	80	77.8	69.2	66.7	79.2	60	73.3	61.5	86.7	87.5	62.5
	*P* value		0.057		0.86				0.91				0.66			0.30			
Can the hemostatic medicament cause any problem or affect the impression material?	Yes	15 (34.1)	23.1	38.7	41.7	50	50	0	50	33.3	23.1	33.3	45.8	0	26.7	7.7	40	37.5	62.5
	*P* value		0.32		**0.033**				0.61				0.11			0.07			
Which technique yields more accurate impression and less incidence of repeating the impression.	Retraction cords with hemostatic agent	21 (47.7)	53.8	45.2	58.3	37.5	50	50	50	44.4	61.5	0	58.3	80	20	30.8	46.7	50	75
	Retraction paste	5 (11.4)	15.4	9.7	0	25	0	8.3	0	5.6	23.1	33.3	4.2	0	26.7	23.1	13.3	0	0
	No difference	18 (40.9)	30.8	45.2	41.7	37.5	50	41.7	50	50	15.4	66.7	37.5	20	53.3	46.2	40	50	25
	*P* value		0.65		0.50				0.14				**0.04**			0.40			
When you do GD with non-vital tooth, do you use local anesthesia:	Yes	42 (95.5)	84.6	100	100	100	100	83.3	100	100	100	33.3	100	100	86.7	84.6	100	100	100
	*P* value		**0.025**		0.13				**0.006**				0.13			0.17			
Retraction paste is easier to use than retraction cord:	Strongly agree	3 (6.8)	7.7	6.5	8.3	6.2	25	0	10	0	15.4	0	4.2	0	13.3	7.7	6.7	0	12.5
	Agree	16 (36.4)	30.8	38.7	25	62.5	0	25	50	33.3	30.8	33.3	37.5	20	40	38.5	40	25	37.5
	Neither agree nor disagree	19 (43.2)	46.2	41.9	58.3	25	75	41.7	30	50	46.2	33.3	45.8	60	33.3	38.5	46.7	62.5	25
	Disagree	6 (13.6)	15.4	12.9	8.3	6.2	0	33.3	10	16.7	7.7	33.3	12.5	20	13.3	15.4	6.7	12.5	25
	*P* value		0.97		0.07				0.76				0.83			0.91			
Retraction paste is less time consuming than retraction cord:	Strongly agree	4 (9.1)	7.7	9.7	8.3	6.2	25	8.3	10	5.6	15.4	0	4.2	20	13.3	15.4	6.7	0	12.5
	Agree	16 (36.4)	23.1	41.9	50	56.2	0	8.3	30	44.4	30.8	33.3	50	0	26.7	23.1	46.7	37.5	37.5
	Neither agree nor disagree	17 (38.6)	46.2	35.5	41.7	18.8	75	50	50	27.8	46.2	33.3	37.5	40	40	38.5	33.3	50	37.5
	Disagree	7 (15.9)	23.1	12.9	0	18.8	0	33.3	10	22.2	7.7	33.3	8.3	40	20	23.1	13.3	12.5	12.5
	*P* value		0.62		0.07				0.87				0.25			0.94			
Retraction paste is more comfortable to the patient than retraction cord:	Strongly agree	6 (13.6)	23.1	9.7	16.7	18.8	25	0	20	11.1	15.4	0	16.7	0	13.3	7.7	13.3	12.5	25
	Agree	19 (43.2)	38.5	45.2	50	50	25	33.3	40	38.9	53.8	33.3	50	20	40	46.2	53.3	25	37.5
	Neither agree nor disagree	17 (38.6)	30.8	41.9	33.3	25	50	58.3	40	44.4	30.8	33.3	29.2	80	40	38.5	33.3	50	37.5
	Disagree	2 (4.5)	7.7	3.2	0	6.2	0	8.3	0	5.6	0	33.3	4.2	0	6.7	7.7	0	12.5	0
	*P* value		0.57		0.67				0.51				0.55			0.84			
Retraction paste is more effective to control bleeding than retraction cord:	Strongly agree	1 (2.3)	0	3.2	0	6.2	0	0	0	0	7.7	0	0	0	6.7	7.7	0	0	0
	Agree	12 (27.3)	30.8	25.8	8.3	37.5	50	25	10	16.7	53.8	33.3	20.8	20	40	30.8	40	12.5	12.5
	Neither agree nor disagree	24 (54.5)	38.5	61.3	66.7	37.5	50	66.7	70	66.7	23.1	66.7	62.5	80	33.3	38.5	53.3	75	62.5
	Disagree	7 (15.9)	30.8	9.7	25	18.8	0	8.3	20	16.7	15.4	0	16.7	0	20	23.1	6.7	12.5	25
	*P* value		0.27		0.53				0.24				0.38			0.57			
Retraction paste is less traumatic to the gingival tissue and cause less recession than retraction cord:	Strongly agree	3 (6.8)	7.7	6.5	8.3	12.5	0	0	0	11.1	7.7	0	8.3	0	6.7	7.7	6.7	12.5	0
	Agree	22 (50)	53.8	48.4	50	62.5	25	41.7	60	33.3	61.5	66.7	54.2	40	46.7	46.2	46.7	37.5	75
	Neither agree nor disagree	16 (36.4)	23.1	41.9	25	25	75	50	40	44.4	23.1	33.3	29.2	60	40	30.8	40	50	25
	Disagree	3 (6.8)	15.4	3.2	16.7	0	0	8.3	0	11.1	7.7	0	8.3	0	6.7	15.4	6.7	0	0
	*P* value		0.40		0.40				0.79				0.90			0.78			
Retraction paste is more cost efficient than retraction cord:	Strongly agree	3 (6.8)	15.4	3.2	0	18.8	0	0	0	11.1	7.7	0	4.2	20	6.7	7.7	13.3	0	0
	Agree	7 (15.9)	23.1	12.9	8.3	25	0	16.7	10	22.2	15.4	0	8.3	0	33.3	15.4	20	12.5	12.5
	Neither agree nor disagree	18 (40.9)	53.8	35.5	50	18.8	50	58.3	60	33.3	30.8	66.7	41.7	40	40	46.2	26.7	50	50
	Disagree	15 (34.1)	7.7	45.2	41.7	31.2	50	25	30	27.8	46.2	33.3	45.8	40	13.3	23.1	40	37.5	37.5
	Strongly disagree	1 (2.3)	0	3.2	0	6.2	0	0	0	5.6	0	0	0	0	6.7	7.7	0	0	0
	*P* value		0.11		0.38				0.87				0.21			0.89			
Retraction paste causes gingival discoloration:	Strongly agree	1 (2.3)	0	3.2	0	6.2	0	0	0	0	7.7	0	0	0	6.7	7.7	0	0	0
	Agree	8 (18.2	15.4	19.4	16.7	18.8	25	16.7	10	5.6	38.5	33.3	20.8	0	3	15.4	20	12.5	25
	Neither agree nor disagree	28 (63.6)	46.2	71	75	50	50	75	80	77.8	30.8	66.7	66.7	100	46.7	61.5	53.3	75	75
	Disagree	7 (15.9)	38.5	6.5	8.3	25	25	8.3	10	16.7	23.1	0	12.5	0	4	15.4	26.7	12.5	0
	*P* value		0.07		0.85				0.22				0.34			0.76			

### Practice of gingival displacement

The majority 144 (76.6%) use GD in their clinics, with significantly lower rates among females compared with males (*P* = 0.046). As shown in Table 2, of those who used the GD, 130 (90.3%), 75 (53.6%), 96 (68.6%), and 34 (24.3%) used it with full coverage indirect restorations, partial coverage indirect restorations, composites, and with impressions for implants, respectively. The use with partial coverage restorations was significantly associated with education and experience. The rate of use of GD with impression for implant was significantly associated with work sector.

Half of those who apply GD use it both with digital and conventional impressions, while a minority use it with only digital impressions. In the case of fixed prostheses, 62 (43.1%), 135 (93.8%), 25 (17.4%), and 39 (27.1%) use GD during the preparation (particularly male dentists), impression, provisional (particularly male dentists), and cementation (particularly those with excellent GPAs) stages, respectively. Most of the dentists 104 (72.2%) used retraction cords alone for GD (particularly interns and postgraduate students (PGS) and those with < 5 years of experience); only 4 (2.8%) used retraction paste alone; and 36 (25%) (particularly specialists and those with > 5 years of experience) used both.

### Techniques of gingival displacement

Among those who use GD, 37 (25.7%) always use the single cord technique (particularly interns and general practitioners (GPs) and those not working currently), 10 ( 6.9%) always use the double cord technique, and the majority 97 (67.4%) use both techniques depending on the case treated (particularly PGS). Regarding the type of cord used, nearly one-third 43 (29.9%) used twisted retraction cord (particularly those with 5–10 years of experience), 39 (27.1%) used knitted type (least by those working in the private sector), and 47 (32.6%) used braided type of retraction cord (particularly specialists and those with excellent GPAs). More than half 83 (57.6%) of participants use impregnated retraction cords, particularly specialists, those with excellent GPAs, and those with > 5 years of experience.

Among those who use GD, 90 (62.5%) soak the retraction cord in a hemostatic medicament before they pack it, particularly those working in the academic sector. The most prevalent hemostatic medicament used was ferric sulfate (particularly by those with < 5 years of experience), followed by aluminum sulfate, aluminum chloride (least by those working in the private sector), aluminum potassium sulfate (particularly by males, specialists, those with > 10 years of experience, and those working in both academic and private sectors), local anesthesia, and epinephrine (particularly by those with < 5 years of experience).

Only 12 (8.3%) have experienced adverse systemic problems using epinephrine as a hemostatic agent, mainly those working in both the academic and private sectors. In contrast, 44 (30.6%) have experienced adverse local tissue problems when using a hemostatic medicament, and 23 (16%) reported that the hemostatic medicament caused some problems or affected the impression material.

Nearly one-quarter 38 (26.4%) use electrosurgery in their clinics to obtain GD and hemostasis, particularly males, specialists, and those with > 5 years of experience.

### Application of retraction pastes

Of those who use retraction paste for GD, the majority use Astringent Retraction Paste (particularly those with 5–10 years of experience), followed by Expasyl, Racegel, Taxodent, GingiTrac, and Access Edge.

When asked which technique yields a more accurate impression and less incidence of repeating the impression, 93 (64.6%) reported that the technique was retraction cords with a hemostatic agent, 17 (11.8%) said retraction cords without a hemostatic agent, 14 (9.7%) said retraction paste, and 20 (13.9%) think that the technique makes no difference. Two-thirds use local anesthesia with retraction cords when they do GD with non-vital teeth; 3 (2.1%) use it with retraction pastes; 15 (10.4%) with both; and 30 (20.8%) with neither.

More than half 75 (52.1%) thought that retraction paste is easier to use, 122 (63.9%) less time consuming, 87 (60.4%) more comfortable for the patient, 44 (30.6%) more effective in controlling bleeding, 97 (67.3%) less traumatic to the gingival tissue and causes less recession, and 30 (20.9%) more cost efficient than retraction cord. Nearly one-quarter 34 (23.6%) think that retraction paste causes gingival discoloration.

### Attitude and knowledge toward gingival displacement techniques

Among 44 dentists who never used GD before, 37 ( 84.1%), 13 ( 29.5%), 21 (47.7%), and 16 (36.4%) think that they need to use GD with full coverage restorations, partial coverage restorations, composite restorations, and impressions for implants, respectively. While 3 (6.8%) thought they should use GD with digital impressions only, 18 (40.9%) thought they should use it with conventional impressions only, and 23 (52.3%) thought they should use it with both impressions. When asked about the method used for GD, 40 (90.9%) thought they can use retraction cord (particularly those with less years of experience), 17 (38.6%) thought they could use retraction paste (particularly interns and GPs), while 10 (22.7%) thought they could use electrosurgery.

Regarding the specific retraction cord technique (single vs. double), 5 (11.4%) (particularly PGS) thought that the single technique is more accurate, 13 (29.5%) (particularly interns) believed the double technique was more accurate, 17 (38.6%) (particularly PGS) select the technique depending on the case, and 9 (20.5%) did not know the difference between the two techniques (particularly specialists).

Out of those who did not use GD before, 2 (45.5%) thought that when epinephrine is used as a hemostatic agent, adverse systemic side effects might occur; on the other hand, 33 (75%) thought that hemostatic medicaments might be associated with adverse local tissue problems, and 15 (34.1%) believed that the hemostatic medicaments do affect the final impression material.

When participants were asked specifically about the technique that yields more accurate results with a lower incidence of repeating the impression, 21 (47.7%) chose the retraction cord with a hemostatic agent (particularly those with 5–10 years of experience), 5 (11.4%) chose the retraction paste (particularly those with > 10 years of experience), and 18 (40.9%) stated that there is no difference between the various GD techniques. However, when GD was performed on non-vital teeth, 42 (95.5%) suggested the use of local anesthesia (females more than males, those with fair GPAs less than those with higher GPAs).

When the perception of those who did not practice GD was evaluated, the data revealed that 19 (43.2%) thought that retraction paste is easier to use; 20 (45.5%) believed it to be less time-consuming; 25 (56.8%) thought it is more comfortable for the patient; 13 (29.6%) declared it more effective in controlling bleeding; 25 (56.8%) thought it is less traumatic to the gingival tissue and would result in less recession; and 10 (22.7%) stated that it is more cost-efficient than retraction cord. Finally, nearly one-fifth 9 (20.5%) think that retraction paste causes gingival discoloration.

## Discussion

Gingival displacement is considered a primordial and mandatory step for most of the restorative procedures that involve close proximity to the gingival sulcus. Improper displacement can be detrimental to the quality of the final restorative work [1–4]. The survey of this research was designed to investigate and study the practice preferences and the general knowledge and attitude of Jordanian dentists toward GD concepts.

According to the results of this survey, 144 (76.5%) of the participants apply GD methods and answered the survey based on their practice and skills. The other 44 (23.5%) revealed that they do not use GD in their clinical practice and provided answers based on their knowledge. One possible reason behind the fact that some participants do not use GD could be that they are not doing restorative work in their practices. They are either periodontists, endodontists, or other specialty practitioners who do not require GD in their daily work or could be general dentists who are not currently practicing. Female participants represented 83 (57.4%) of the sample size, which is a little more than male participants. This is not surprising as the female-to-male dentists’ ratio is high in Jordan. The majority of participants were specialists and general dentists with more than 10 years of experience and a very good GPA. That could be explained by the fact that those individuals are the most confident answering this survey.

Based on the participants’ practice and knowledge, GD was mostly needed with full-coverage restorations. Participants with higher education apparently use GD with partial-coverage restorations more frequently than the other participant categories. In fact, partial coverage restorations are more technique-sensitive and might be performed more frequently by specialists than GPs.

Most participants reported that they use GD concurrently with impression-making in fixed prosthodontic work. Surprisingly, only half of the participants reported using GD with both conventional and digital impressions. Nonetheless, it is well known that the GD is an essential step for both impression techniques [18].Almost two-third of participants reported using retraction cords for GD, while specialists and experienced dentists reported the use of both retraction cords and pastes in their practices. This could be attributed to the fact that specialists have a higher flow of advanced cases in which they might need both techniques to get enough GD and control bleeding. Only a minority of participants reported that they only use retraction pastes. Those findings suggest that the cordless practice is not common in Jordan.

The types of retraction cords used by participants varied between twisted, knitted, and braided. More than half of the participants use impregnated retraction cords or soak the cord in hemostatic medicament. This technique helps control the bleeding from gingival tissues, facilitates the impression procedure, and ensures a more accurate final outcome [2].

From another aspect and regarding the side effects associated with GD, only 12 (8%) of participants reported noticing adverse systemic side effects related to the use of epinephrine. An interesting and coherent finding, since epinephrine’s side effects are well documented in the literature [17]. However, a higher percentage of participants reported adverse effects on gingival tissues related to the hemostatic medicaments, while a substantial number reported encountering problems related to the impression itself following gingival displacement.

Regardless of the fact that half of the participants reported retraction pastes being easier to use, less time-consuming, more comfortable for the patient, and causing less trauma to the gingival tissues, 93 (64.6%) of them stated that the impression is more predictable with the use of retraction cords impregnated with hemostatic medicament. In summary, it is necessary to acknowledge that the gingival displacement method should be done cautiously in order to achieve the best outcome while causing the least trauma to the gingival tissues and ensuring high patient satisfaction.

Reviewing and appraising the knowledge of participants who do not use GD in their practice is a noteworthy part of the survey. Our results showed that participants realize that GD is required for full coverage restorations as well as composite restorations with subgingival margins. Furthermore, more than half the participants understand that GD is needed for both conventional and digital impressions. Around one-third believed that the choice of single vs. double cord technique was case-dependent. The fact that 20 (45%) of participants know that epinephrine can result in systemic side effects and 33 (75%) believe that hemostatic medicaments can cause gingival damage indicates that the knowledge of the participants is satisfactory and comparable to what is reported in the literature [2, 17]. The most predictable impression technique based on participants’ knowledge was retraction cords impregnated with hemostatic agents, which was comparable to the answers based on practice and skill. Fewer participants believed that retraction pastes are sometimes easier to use, less time-consuming, more comfortable for the patient, and cause less trauma to the gingival tissues compared to the group who answered based on their practice. This conclusion might be related to the fact that the former participants are not familiar with these materials and do not possess adequate experience or knowledge concerning their use.

Based on the results of this survey, a comprehensive continuing education course explaining the new GD methods and comparing them with the conventional mechanical methods is advisable. Case selection is one of the most important factors that determine which technique is best suited to a specific clinical situation.

## Conclusion

The cordless GD technique is believed to be easier, faster, and less traumatic to the gingival tissues; nevertheless, the outcome of dental impressions is believed to be more predictable with the use of conventional retraction cords and hemostatic medicaments.

### Supplementary Information

**Additional file 1.**

## Data Availability

All data are available upon request from the corresponding author ( i.abdalraheam@ju.edu.jo).
