# Supplementary material for: Dentists’ knowledge and preference regarding gingival displacement methods
Source: BMC Oral Health. 2023 Aug 16;23:574. doi: 10.1186/s12903-023-03218-1 (PMC10429067; doi:10.1186/s12903-023-03218-1)
Supplement: Supplementary file 1 — Additional file 1. [file 12903_2023_3218_MOESM1_ESM.docx]

**Additional file 1**

Dentists’ knowledge and preference regarding gingival displacement techniques (cords vs pastes)

Section 1

This study does not require any personal information about you (name, ID number, or anything else), so we hope that you will answer all questions absolutely freely without hesitation or fear.
All results will be treated confidentially and will not be viewed by any party outside the search group.
Your accurate and transparent answers will greatly contribute to the success of this research and the treatment of any problems raised.
We thank you for giving us a valuable part of your time to complete this survey.

Section 2

Gender

- Male
- Female

Level of education:

- Intern
- General practitioner
- Postgraduate student / resident
- Specialist

Cumulative GPA

- Excellent (90-100%)
- Very good (80-89%)
- Good (70-79%)
- Fair (60-69%)
- Poor (<60%)

Year of experience

- <5 years
- 5-10 years
- >10 years

Dental Degree

- General practitioner
- Specialist

Practice type

- Academic practice
- Private practice
- Both
- Inter (don't work)

Section 3

Gingival displacement

1) Do you use any type of gingival displacement in your clinic?

- Yes
- No

Section 4

Practice toward gingival displacement techniques

Regarding the next questions, please answer the following questions based on your experience and knowledge.

1) What is/are the type/s of restorations that you use gingival displacement with it/them?

- Full coverage indirect restoration (crown, PFM, ceramic)
- Partial coverage indirect restoration (onlay, inlay, venner, etc.)
- Composite direct restoration
- Impression for implant
- Other

2) What is/are the type/s of impression that you use gingival displacement with it/them?

- Digital impression
- Conventional impression
- Both

3) Regarding the fixed prostheses, what is/are the step/s that you use gingival displacement with it/them?

- Preparation
- Impression
- Provisional
- Cementation

4) Which type of gingival displacement technique do you use?

- Retraction cord
- Cordless (retraction paste)
- Cord and paste

Section 5

1) What is the technique of retraction cord do you use?

- Single technique
- Double technique
- Both (depends on the case)

2) What is the technique of retraction cord do you use?

- Twisted
- Knitted
- Braided
- I do not know

3) Do you use impregnated retraction cord?

- Yes
- No
- I do not know

4) Do you soak the retraction cord in a hemostatic medicament before you pack it?

- Yes
- No

5) What is/are the type/s of the hemostatic medicament that you use with the retraction cord?

- Epinephrine
- Aluminum potassium sulfate
- Aluminum chloride
- Aluminum sulphate (hemodent)
- Ferric sulphate (viscostat)
- Local anesthesia
- Other

6) Have you experienced any adverse systemic problems using epinephrine as a hemostatic agent?

- Yes
- No
- I do not use it

If the answer was yes check appropriate symptom/s:

- Increased pulse rate
- Cold sweat
- Anxiety
- Syncope
- Cardiac arrest
- Increased blood pressure
- Other

7) Have you experienced any adverse local tissue problems when using a hemostatic medicament?

- Yes
- No
- I do not use it

If the answer was yes check appropriate symptom/s:

- Tissue inflammation
- Tissue discoloration
- Tissue sloughing
- Recession
- Other

8) Did the hemostatic medicament cause any problem or affect the impression material?

- Yes
- No
- I do not use it

9) Do you use electrosurgery in your clinic to obtain gingival displacement and hemostasis?

- Yes
- No

Section 6

Retraction paste

What is/are the type/s of retraction paste that you use?

- Expasyl (Kerr)
- Racegel (Septodont)
- Traxodent (Premier)
- GingiTrac (Centrix)
- Access Edge (Centrix)
- Astringent Retraction Paste (3M)
- Other
- I don’t use any

Section 7

1) Which technique yields more accurate impression and less incidence of repeating the impression?

- Retraction cords with hemostatic agent
- Retraction cords without hemostatic agent
- Retraction paste
- No difference

2) When you do gingival displacement with non-vital tooth, do you use local anesthesia with:

- Retraction cord
- Retraction paste
- BOTH
- Neither

3) Retraction paste is easier to use than retraction cord:

- Strongly agree
- Agree
- Neither agree nor disagree
- Disagree
- Strongly disagree

4) Retraction paste is less time consuming than retraction cord:

- Strongly agree
- Agree
- Neither agree nor disagree
- Disagree
- Strongly disagree

5) Retraction paste is more comfortable to the patient than retraction cord:

- Strongly agree
- Agree
- Neither agree nor disagree
- Disagree
- Strongly disagree

6) Retraction paste is more effective to control bleeding than retraction cord:

- Strongly agree
- Agree
- Neither agree nor disagree
- Disagree
- Strongly disagree

7) Retraction paste is less traumatic to the gingival tissue and cause less recession than retraction cord:

- Strongly agree
- Agree
- Neither agree nor disagree
- Disagree
- Strongly disagree

8) Retraction paste is more cost efficient than retraction cord:

- Strongly agree
- Agree
- Neither agree nor disagree
- Disagree
- Strongly disagree

9) Retraction paste causes gingival discoloration:

- Strongly agree
- Agree
- Neither agree nor disagree
- Disagree
- Strongly disagree

Section 8

Knowledge toward gingival displacement techniques

Regarding the next questions, please answer the following questions based on your knowledge.

1) What is/are the type/s of restorations that you need to use gingival displacement technique with it/them?

- Full coverage indirect restoration (crown, PFM, ceramic)
- Partial coverage indirect restoration (onlay, inlay, venner, etc.)
- Composite direct restoration
- Impression for implant
- Other

2) What is the type of impression that you need to use gingival displacement techniques with it?

- Digital impression
- Conventional impression
- Both

3) What is/are the type/s of gingival displacement technique that you can use it/them?

- Retraction cord
- Cordless (retraction paste)
- Electrosurgery

4) Regarding the retraction cord techniques (single vs double), which technique is more accurate?

- Single technique
- Double technique
- Depends on the case
- I do not know the difference between them

5) Regarding the retraction cord types (impregnated vs non-impregnated), which technique is better?

- Impregnated
- Non-impregnated
- Depends on the case
- I do not know the difference

6) What is/are the type/s of the hemostatic medicament that you can use with the retraction cord?

- Epinephrine
- Aluminum potassium sulfate
- Aluminum chloride
- Aluminum sulphate (hemodent)
- Ferric sulphate (viscostat)
- Local anesthesia
- Other

7) Are there any adverse systemic side effects that can happen when using epinephrine as a hemostatic agent?

- Yes
- No

If the answer was yes check appropriate symptom/s:

- Increased pulse rate
- Cold sweat
- Anxiety
- Syncope
- Cardiac arrest
- Increased blood pressure
- Other

8) Are there any adverse local tissue problems that can happen when using a hemostatic medicament?

- Yes
- No

If the answer was yes check appropriate symptom/s:

- Tissue inflammation
- Tissue discoloration
- Tissue sloughing
- Recession
- Other

9) Can the hemostatic medicament cause any problem or affect the impression material?

- Yes
- No

10) What is/are the type of retraction paste/s that you can use?

- Expasyl (Kerr)
- Racegel (Septodont)
- Traxodent (Premier)
- GingiTrac (Centrix)
- Access Edge (Centrix)
- Astringent Retraction Paste (3M)
- Other
- I don’t know any of them

11) Which technique yields more accurate impression and less incidence of repeating the impression?

- Retraction cords with hemostatic agent
- Retraction cords without hemostatic agent
- Retraction paste
- No difference

12) When you do gingival displacement with **non-vital tooth, do you use local anesthesia with:**

- Retraction cord
- Retraction paste
- BOTH
- Neither

13) Retraction paste is easier to use than retraction cord:

- Strongly agree
- Agree
- Neither agree nor disagree
- Disagree
- Strongly disagree

14) Retraction paste is less time consuming than retraction cord:

- Strongly agree
- Agree
- Neither agree nor disagree
- Disagree
- Strongly disagree

15) Retraction paste is more comfortable to the patient than retraction cord:

- Strongly agree
- Agree
- Neither agree nor disagree
- Disagree
- Strongly disagree

16) Retraction paste is more effective to control bleeding than retraction cord:

- Strongly agree
- Agree
- Neither agree nor disagree
- Disagree
- Strongly disagree

17) Retraction paste is less traumatic to the gingival tissue and cause less recession than retraction cord:

- Strongly agree
- Agree
- Neither agree nor disagree
- Disagree
- Strongly disagree

18) Retraction paste is more cost efficient than retraction cord:

- Strongly agree
- Agree
- Neither agree nor disagree
- Disagree
- Strongly disagree

19) Retraction paste causes gingival discoloration:

- Strongly agree
- Agree
- Neither agree nor disagree
- Disagree
- Strongly disagree
